# Response to a Water Bolus in Long Term Oral Contraceptive Users

**DOI:** 10.3389/fspor.2022.857719

**Published:** 2022-04-25

**Authors:** Whitley C. Atkins, Brendon P. McDermott, Abigail T. Colburn, Stavros A. Kavouras

**Affiliations:** ^1^Exercise Science Research Center, Department of Health and Human Performance, Fayetteville, AR, United States; ^2^Hydration Science Laboratory, Arizona State University, Tempe, AZ, United States

**Keywords:** euhydration, female sex hormones, copeptin, hormonal contraception, resting conditions

## Abstract

The purpose of our study was to determine the responses to an acute water bolus in long-term oral contraception (OCP) users. Seventeen female volunteers (27 ± 5 y, 64.1 ± 13.7 kg, 39.6 ± 5.9 kg/LBM) provided consent and enrolled in our study. All were long-term OCP users and participated in two trials, one during the active pill (High Hormone, HH) dose of their prescribed OCP and one during the sham pill (Low Hormone, LH) dose. Participants reported to the laboratory euhydrated, were fed breakfast, remained seated for 60 min and were provided a bolus of room temperature water in the amount of 12 mL/kg/LBM. Urine output over 180 min was measured. Nude body mass was measured pre- and post-trial. Urine specific gravity (USG) and urine osmolality were analyzed. Between trials, there were no differences in 3-h total urine volume (*P* = 0.296), 3-h USG (*P* = 0.225), 3-h urine osmolality (*P* = 0.088), or 3-h urine frequency (*P* = 0.367). Heart rate was not different between trials (*P* = 0.792) nor over time (*P* = 0.731). Mean arterial pressure was not different between trials (*P* = 0.099) nor over time (*P* = 0.262). Perceived thirst demonstrated a significant main effect for increasing over time regardless of trial (*P* < 0.001) but there was no difference between trials (*P* = 0.731). The urgency to void was not different between trials (*P* = 0.149) nor over time (*P* = 0.615). Plasma volume change was not different between trials (*P* = 0.847) (HH: −3.4 ± 5.0, LH post: −3.8 ± 4.5%) and plasma osmolality did not differ between trials (*P* = 0.290) nor over time (*P* = 0.967) (HH pre: 290 ± 4, HH post: 289 ± 4, LH pre: 291 ± 4, LH post: 291 ± 4 m_osm_/L). Blood glucose significantly decreased over time (*P* < 0.001) but there was no difference between trials (*P* = 0.780) (HH pre: 95.9 ± 113.9, HH post: 86.8 ± 6.5, LH pre: 95.9 ± 13.5, LH post: 84.6 ± 9.4 mmol/L). Copeptin concentration did not differ between phases of OCP use (*P* = 0.645) nor from pre- to post-trial (*P* = 0.787) Despite fluctuations in hormone concentrations, responses to a water bolus seem to be unaffected in OCP users in euhydrated, resting conditions.

## Introduction

The benefits of optimal hydration include enhanced mood maintenance, cognitive function, recovery from exercise, heat dissipation and athletic performance (McDermott et al., 2017). It is established that long-term health, including glucose regulation, is positively influenced by proper hydration (Manz and Wentz, 2005; Popkin et al., 2010; Roncal-Jimenez et al., 2015; Jansen et al., 2019; Stookey et al., 2020). In order to meet hydration needs, the importance of individual considerations has been proposed (Sawka et al., 2005; McDermott et al., 2017; Armstrong and Johnson, 2018). However, sex difference considerations beyond body-size and sweat rate have not been established.

Female sex hormones, predominantly estrogen and progesterone, impact fluid balance by interacting with hormones responsible for fluid regulation (Stachenfeld et al., 1999; Calzone et al., 2001; Stachenfeld and Taylor, 2005; Stachenfeld, 2008; Giersch et al., 2020). Circulating arginine vasopressin (AVP) is positively correlated with estrogen concentrations (Stachenfeld et al., 1999; Stachenfeld and Keefe, 2002; Giersch et al., 2021). Further, high estrogen concentrations decrease the osmotic threshold needed for AVP release (Stachenfeld et al., 1999; Stachenfeld and Keefe, 2002; Giersch et al., 2021). Concomitantly, progesterone may affect fluid balance *via* aldosterone pathways due to mineralocorticoid receptor competition, although progesterone's role in fluid balance is not fully understood (Myles and Funder, 1996; Giersch et al., 2021). These interactions complicate expert recommendations for individualized fluid intake recommendations without specific knowledge of the extent of fluid balance differences.

Unfortunately, it has become common practice to exclude females in fluid balance research; noting that there are differences due to menstrual cycle phase. Although hormonal influence on fluid balance is acknowledged, methodological design often does not account for variations in hormones across the menstrual cycle (Sims and Heather, 2018; Elliott-Sale, 2021). This results in females being excluded entirely from fluid balance studies or only participating during the follicular phase of the menstrual cycle, when circulating sex hormones that may influence fluid balance are at their lowest concentrations. This practice prevents scientific physiological advances for females. Testing during the follicular phase accounts for a small portion of the menstrual cycle (<1/3) and does not account for those using hormonal contraception.

According to the National Center for Health Statistics data from the 2015 to 2017 National Survey of Family Growth, 64.9% of the 72.2 million females aged 15–49 in the U.S were using contraception (Daniels and Abma, 2018). Of the reversible methods of contraception, the most common was the use of oral contraceptive pills (12.6%; OCPs) within the previously mentioned age range (Daniels and Abma, 2018). Stachenfeld (2008) further reported that >90% of European and U.S. females are currently taking or previously have taken OCPs. In athletes, the prevalence of females taking combined, monophasic OCPs is one in two (50%) (Lei et al., 2019). Fluid balance studies have suggested that OCPs containing estrogen (combined hormone OCPs) increased osmotically induced AVP and thirst during dehydration and *ad libitum* rehydration even though there were no changes in water retention (Stachenfeld et al., 1999); confirming that estrogen decreases the plasma osmotic threshold.

To date, there are limited data on the effects of OCP use on fluid balance. A better understanding of female physiology may improve sport and work performance (Charkoudian and Stachenfeld, 2016). Therefore, the purpose of our study was to determine the acute responses to a water bolus in long-term oral contraceptive users. To our knowledge, this study represents the first investigation of a given water bolus in OCP users and may be viewed as an initial step in the investigations of OCP use on fluid balance and hydration.

## Methodology

### Ethics Approval

The experimental protocol was reviewed and approved by University of Arkansas Institutional Review Board. All participants were taken through and signed an informed consent form prior to taking part in this study. All data were collected in accordance with the Declaration of Helsinki.

### Subjects

Seventeen females voluntarily participated in this study. Participant characteristics are shown in Table 1. All participants were long-term OCP users, defined as ≥ 6 months of prescribed use. Only combined (estradiol and progestin), monophasic OCPs were included. Following medical clearance determined by medical history questionnaire, participants were briefed on trial procedures and signed the IRB-approved informed consent form. Individuals were excluded if they reported a history of cardiac, renal, respiratory or major organ pathology, or were taking supplements or medications known to alter fluid balance.

**Table 1:** Participant characteristics.

***n***	**Age (y)**	**Weight (kg)**	**BMI**	**LBM (kg)**	**Body fat (%)**
17	27 ± 5	64.1 ± 13.7	24.7 ± 5.3	39.6 ± 5.9	35.5 ± 6.8

*Data presented as Mean ± SD*.

### Procedures

We employed a crossover, repeated measures design to evaluate the effects of OCP use on a given water bolus in resting conditions. Participants reported to the exercise science research center for a total of five visits: a familiarization visit, two pre-trial visits to pick up materials, and two experimental trials. Experimental trials were scheduled so that the participant was on the active pill dose of prescribed OCPs for one trial (high hormone, HH) and on the placebo or sham dose for the other trial (low hormone, LH) for at least 48 h. Trials were conveniently scheduled, but balanced (eight participants completed the HH trial prior to the LH trial). All trials were conducted at the same time of day to control for circadian variation.

### Familiarization Visit

Once medically cleared, participants had their descriptive information recorded. Body height was assessed using a stadiometer and body mass with a calibrated scale (Model 379X; Health-o-meter, Chicago, IL). Body fat % and body surface area were assessed *via* dual-energy X-ray absorption (Lunar Prodigy; General Electric, Boston, MA; DXA) scan. Participants were familiarized with perceptual scales for perceived thirst and sense of urgency. Participants were instructed on participation duties and were scheduled for pre-trial visits and experimental trials.

### Pre-trial Visit

Participants reported to the research center 24-h prior to experimental trials. Participants were provided a 24-h standard food and fluid log and urine collection jugs to collect all urine output for 24-h preceding trials. Participants were instructed to draw a line at the fluid line and record the time of day on the urine containers with a permanent marker each time they voided into the container. Participants were asked to avoid moderate to vigorous exercise and alcohol consumption for 24-h and caffeine consumption for 12-h leading into trials. Participants were asked to avoid any food or drink (other than water) consumption for 8-h leading into the trials. Participants were provided with and instructed to consume an extra 473 mL (16oz) of water prior to sleeping and upon waking on trial days. Water provisions were provided to ensure participants arrived euhydrated prior to data collection. Compliance was confirmed with the use of a 24-h self-report sheet upon arrival to trials. Pill dosing was confirmed *via* visual confirmation of pill-pack.

### Experimental Trials

Participants arrived at the research center at 7 am and were fed a standard breakfast consisting of a bagel and either cream cheese or peanut butter and almonds. Breakfast was weighed and matched within participants. Participants gave their 24-h urine collection jugs and diet and fluid logs to researchers. Urine specific gravity (USG), urine osmolality and 24-h urine volume were measured. Researchers recorded each time of void, sense of urgency and perceived thirst as dictated on the 24-h urine containers. After breakfast, participants were asked to remain seated for 60 min. During this time, a blood sample was collected. After 60 min, participants were asked to void their bladder and a nude body mass was measured and recorded. Once seated again, participants consumed a room-temperature bolus of water in the amount of 12 mL/kg of lean body mass (LBM) provided over 20 min in four equal boluses (3 mL/kg/LBM) every 5 min. The volume of water bolus provided in our study was based on previous data by Claybaugh et al. (2000). The amount used in the prior study was appropriate to evoke responses in urine flow rate, plasma arginine vasopressin (whereas copeptin is a surrogate) and percent bolus excreted between phases of eumenorrheic females (Claybaugh et al., 2000). The amount we chose was a reasonable amount that a person would consume in the time allotted and would not maximally dilute urine. Participants were then donned with electrodes and a blood pressure cuff for continuous heart rate and blood pressure measurements (SunTech Tango M2, SunTech Medical, Inc., Morrisville, NC). Perceptual measures (thirst and urgency), heart rate, and blood pressure were recorded 15 min post-fluid ingestion and continued every 30 min for a total of 180 min (3 h) while the participant remained seated. Thirst was assessed by asking the participant, “How thirsty do you feel now?” Participants pointed to the number on a 9-pt scale that corresponded to their current perception of thirst ranging from “1-Not Thirsty At All” to “9-Very, Very Thirsty” (Maresh et al., 2001). Urgency to Void was assessed by asking the participant, “What is your sense of urgency to void now?” Participants pointed to the number on a 5-pt scale that corresponded to their sense of urgency ranging from “0-no sensation” to “4-uncomfortable urge (Tucker et al., 2016).” Participants were free to go to the bathroom when needed. Each voluntary void was counted toward our frequency measure during the 3-h timeframe so that 1 voluntary void during the 3-h was a frequency equal to 1. At 180 min post-ingestion participants were asked to provide a forced void regardless of their sense of urgency, this void was not included in “total frequency.” Each urine void during the 3-h was analyzed separately for USG, osmolality, and volume and then combined for a total assessment. Our time points for thirst, urgency to void, blood pressure and heart rate included 15, 45,75, 105, 135, 165, and 180 min after water ingestion. Upon completion of the trial, nude body mass was measured, and a final blood sample was collected.

### Analyses

Urine samples were analyzed for USG *via* refractometry (Master-Sur; Atago, Tokyo, Japan). Urine osmolality was analyzed *via* freezing point depression following manufacturer guidelines (Model 3250; Advanced Instruments, Andover, MA).

After blood was collected *via* antecubital vein, pre- and post-trial blood samples were analyzed for hemoglobin (HemaCue HB^+^, Angelhom, Sweden) in duplicate and hematocrit (Damon/IEC Division, Needham, MA) in triplicate and used to quantify plasma volume change (Dill and Costill, 1974). Plasma samples collected in lithium heparin tubes were centrifuged (Thermo Scientific, Heraeus Megafuge 16R, Osterode am Harz, Germany) at 1,000 rpm for 15 min immediately post-blood draw and analyzed for plasma osmolality (Model 3250; Advanced Instruments, Andover, MA). Plasma was aliquoted and stored frozen (−80°C) for later analyses of plasma electrolytes (EasyElectrolytes, Medica, Bedford MA), and plasma glucose (YSI 2900 Biochemisty Analyzer, Xylem, Yellow Springs, OH).

Serum samples were permitted to clot for 30 min at room temperature then centrifuged at 1,000 rpm for 20 min, aliquoted and stored frozen (−80°C). Serum copeptin concentration (used as a surrogate for AVP; Jansen et al., 2019; Kavouras, 2019) was analyzed from single samples using immunofluorescent Copeptin proAVP KRYPTOR assay *via* the fully automated random-access immunoassay KRYPTOR Compact PLUS analyzer (BRAHMS, Thermo Fisher, Hennigsdort, Germany).

### Statistical Analyses

Data were reviewed and analyzed for normality and homogeneity of variance. Data were tested using paired samples *t*-tests (two-tailed) and two-way repeated measures analysis of variance (phase × time). If sphericity of data was violated, we interpreted significance according to Greenhouse-Geisser correction. When significant main effect or interactions were identified, *post hoc* comparisons were completed with Bonferroni adjustments due to multiple time point comparisons. Data reported as mean ± SD unless noted. Our significance interpretation was established as *P* ≤ 0.05 and jamovi 2 was used to analyze our data.

## Results

### Pre-trial

There were no significant differences in kcal (HH: 2,165 ± 624, LH: 1,983 ± 835 kcal, *P* = 0.480), carbohydrate (HH: 250 ± 86, LH: 222 ± 91 g, *P* = 0.363), protein (HH: 93 ± 37, LH: 222 ± 91 g, *P* = 0.148), fat (HH: 87 ± 37, LH: 131 ± 44 g, *P* = 0.440), sodium (HH: 3,735 ± 1,770 mg, LH: 3,306 ± 1,951 mg, *P* = 0.512), or potassium (HH: 1,632 ± 1,012, LH: 1,346 ± 737 mg, *P* = 0.360) intake in the 24-h preceding trials. There were no significant differences in 24-h urine volume (HH: 1,564 ± 594, LH: 1,538 ± 588 L, *P* = 0.855), 24-h USG (HH: 1.012± 0.005, LH: 1.012 ± 0.005, *P* = 0.970), 24-h urine osmolality (HH: 463 ± 206, LH:458 ± 178 m_osm_/L, *P* = 0.876) or 24-h urine frequency (HH: 7 ± 2, LH: 7 ± 2, *P* = 0.470) between trials.

### Hydration Status

There was no significant difference in pre-trial spot USG (HH: 1.010 ± 0.006, LH: 1.012 ± 0.009, *P* = 0.478) or pre-trial urine osmolality (HH: 355 ± 275, LH: 419 ± 327 m_osm_/L, *P* = 0.876).

### 3-h Measures

**Figure 1:**
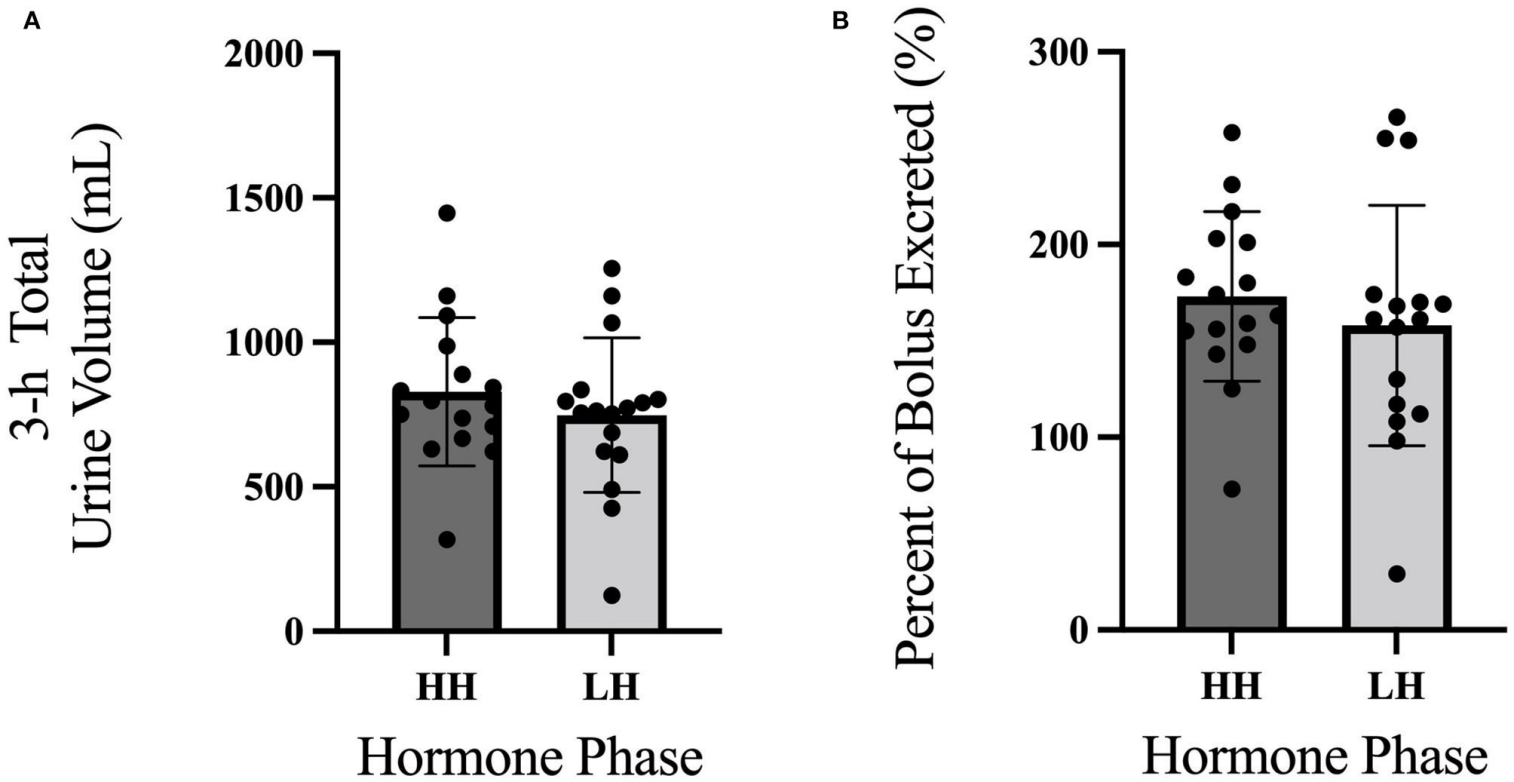
3-h urine volume responses to an acute water bolus. **(A)** Between trials, there were no differences in 3-h total urine volume (*P* = 0.296). **(B)** There was no difference in the percentage of bolus excreted over 3-h between trials (*P* = 0.104).

**Figure 2:**
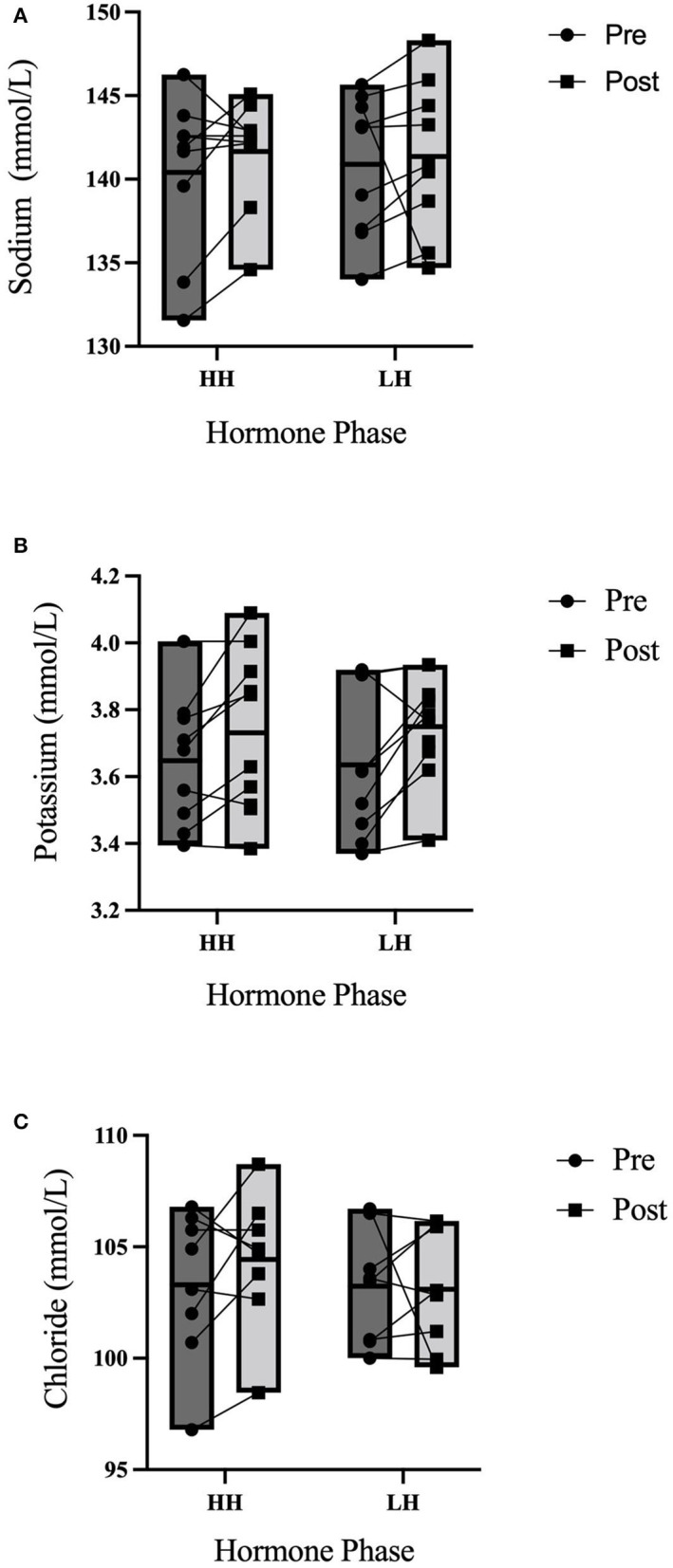
3-h electrolyte responses to an acute water bolus. Individual responses and group summary data is shown (*n* = 9). Markers represented individual responses whereas bars represent group means and range. **(A)** Plasma Na^+^ did not differ from pre- to post-trial (*P* = 0.408) nor were concentration levels different between trials (*P* = 0.275) nor was there an interaction effect (*P* = 0.481). **(B)** There were no differences in Plasma K^+^ over time (*P* = 0.103) or between trials (*P* = 0.852) nor was there an interaction effect (*P* = 0.828). **(C)** There were no differences in Cl^−^ over time (*P* = 0.380), between trials (*P* = 0.428) nor was there an interaction effect (*P* = 0.548).

Between trials, there were no differences in 3-h total urine volume or percent bolus excreted as shown in Figure 1. There were no differences in 3-h USG (*P* = 0.225), 3-h urine osmolality (*P* = 0.088), or 3-h urine frequency (*P* = 0.367) as shown in Table 2. Heart rate was not different between trials (*P* = 0.792) nor over time (*P* = 0.731) nor was there an interaction (*P* = 0.825). Mean arterial pressure was not different between trials (*P* = 0.099) nor over time (*P* = 0.262) nor was there an interaction (*P* = 0.702). Perceived thirst demonstrated a significant main effect for increasing over time regardless of trial (*P* < 0.001) but there was no difference between trials (*P* = 0.731) nor an interaction effect (*P* = 0.164). The sense of urgency to void was not different between trials (*P* = 0.149) nor over time (*P* = 0.615) nor was there an interaction effect (*P* = 0.877). Heart rate, mean arterial pressure, perceived thirst and sense of urgency data are shown in Table 3. Hematocrit increased significantly from pre-water bolus consumption to the 3-h timepoint (*P* = 0.003) but was not different between trials (*P* = 0.754) nor was there an interaction effect (*P* = 0.788) (HH pre: 41 ± 1, HH post: 42 ± 2, LH pre: 41 ± 1, LH post: 42 ± 1%). Hemoglobin significantly increased from pre- water bolus consumption to the 3-h timepoint (*P* = 0.009) but was not different between trials (*P* = 0.309) nor was there an interaction effect (*P* = 0.885) (HH pre: 13.6 ± 0.5, HH post: 13.9 ± 0.6, LH pre: 13.4 ± 0.6, LH post: 13.6 ± 0.5 g/dl). Plasma volume change was not different between trials (*P* = 0.847) (HH: −3.4 ± 5.0, LH post: −3.8 ± 4.5 %). Plasma osmolality did not differ between trials (*P* = 0.337) or over time (*P* = 0.944) nor was there an interaction effect (*P* = 0.725) (HH pre: 290 ± 4, HH post: 290 ± 4, LH pre: 291 ± 5, LH post: 292 ± 2 m_osm_/L). Blood glucose significantly decreased over time (*P* < 0.001) but there was no difference between trials (*P* =0.458) nor was there an interaction effect (*P* = 0.780) (HH pre: 95.9 ± 13.9, HH post: 86.8 ± 6.5, LH pre: 95.9 ± 13.5, LH post: 84.6 ± 9.4 mmol/L. There were no differences in plasma electrolyte changes as shown in Figure 2. Nude body mass demonstrated a main effect for decreasing over time (*P* < 0.001) but did not differ between trials (*P* = 0.993) nor was there an interaction effect (*P* = 0.942) (HH pre: 64.2 ± 14.4, HH post: 63.8 ± 14.2, LH pre: 64.2 ± 14.0, LH post: 63.8 ± 13.8 kg).

**Table 2:** 3-h urinalysis.

	**Urine volume (mL)**	**USG**	**Urine osmolality**	**Urine**
			**(mOsm/L)**	**frequency**
High hormone	829 ± 257	1.005 ± 0.001	169 ± 76	1 ± 1
Low hormone	746 ± 276	1.006 ± 0.004	228 ± 145	2 ± 1

*Data presented as Mean ± SD*.

**Table 3:** 3-h repeated measurements.

		**15-min**	**45-min**	**75-min**	**105-min**	**135-min**	**165-min**	**180-min**
Heart rate (bpm)	High hormone	74 ± 10	74 ± 11	72 ± 10	72 ± 11	70 ± 8	70 ± 9	71 ± 9
	Low hormone	72 ± 8	70 ± 10	71 ± 11	69 ± 10	69 ± 8	73 ± 11	70 ± 10
Systolic blood pressure (mmHg)	High hormone	115 ± 11	119 ± 11	119 ± 11	113 ± 17	112 ± 15	115 ± 14	114 ± 11
	Low hormone	113 ± 12	115 ± 12	115 ± 12	110 ± 10	111 ± 10	113 ± 9	113 ± 9
Diastolic blood pressure (mmHg)	High hormone	70 ± 10	69 ± 12	67 ± 9	62 ± 11	68 ± 12	68 ± 9	71 ± 7
	Low hormone	67 ± 6	69 ± 9	68 ± 7	68 ± 8	68 ± 8	70 ± 8	70 ± 8
Mean arterial pressure (mmHg)	High hormone	85 ± 8	80 ± 9	82 ± 7	79 ± 9	83 ± 11	84 ± 9	85 ± 8
	Low hormone	83 ± 6	84 ± 9	83 ± 7	82 ± 6	82 ± 7	84 ± 9	84 ± 8
Perceived thirst	High hormone	3 ± 1	3 ± 2	3 ± 2	4 ± 2	4 ± 2	4 ± 2	5 ± 2
	Low hormone	2 ± 1	3 ± 2	3 ± 1	3 ± 1	4 ± 1	4 ± 2	4 ± 2
Urgency to void	High hormone	1 ± 1	1 ± 1	1 ± 1	1 ± 1	1 ± 1	1 ± 1	1 ± 1
	Low hormone	1 ± 1	1 ± 1	1 ± 1	1 ± 1	1 ± 1	1 ± 1	1 ± 1

*Data presented as Mean ± SD*.

### Copeptin

**Figure 3:**
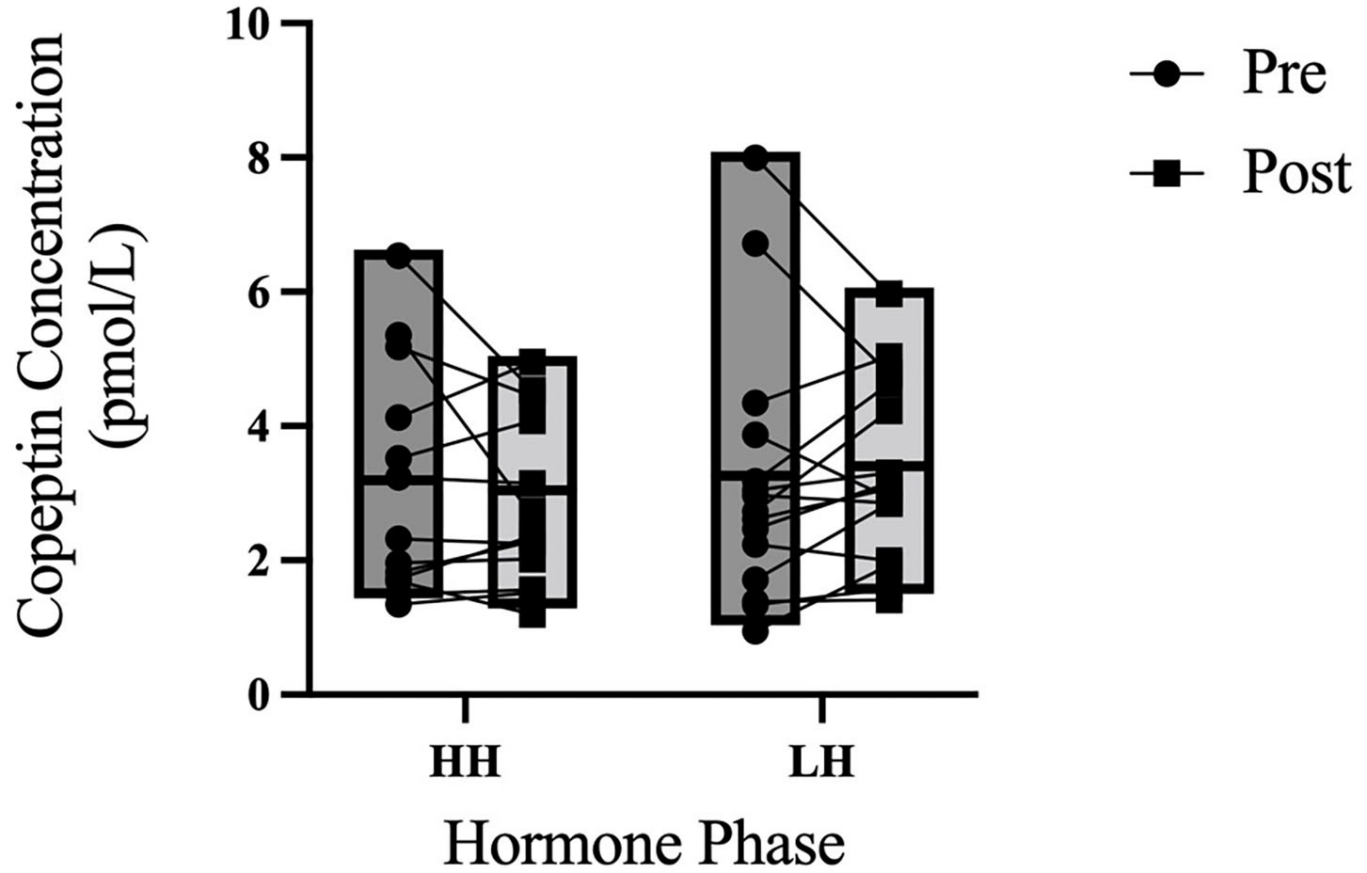
3-h copeptin responses to an acute water bolus. Individual responses and group summary data is presented in Figure 1. Copeptin concentration did not differ between phases of OCP use (*P* = 0.645) nor from pre- to post-trial (*P* = 0.787) nor was there an interaction effect (*P* = 0.333).

Copeptin concentrations are shown in Figure 3.

## Discussion

We examined the physiological effects of an acute standardized water bolus in female, long-term OCP users to provide guidance for female participant inclusion in fluid balance studies. Our findings demonstrate that OCP phase (HH versus LH) does not affect our measured physiological responses to a given bolus in resting conditions. This is the initial study comparing HH and LH phases of OCP use in response to a given water bolus in resting conditions. Our data show no significant differences in the physiological responses measured (thirst, urgency, urine volume, urine osmolality, USG, plasma volume change, electrolytes, and copeptin) to a water bolus between phases of OCP uses.

With the large prevalence of OCP use, it is pertinent for researchers to explore highly applicable physiological questions to the female demographic. OCP use increases circulating concentration of exogenous hormones (estradiol and progestin) compared to endogenous hormones (estrogen and progesterone) in eumenorrheic females. Different brands and types of OCP use make it difficult to control for in research design (Giersch et al., 2020). These reasons have deemed it acceptable to exclude females from sport, exercise, and hydration research citing that females are excluded due to the difficulty in studying them (Sims and Heather, 2018). Whereas, hydration recommendations have been applied to the population even when investigations have solely been completed in males (Giersch et al., 2020). Females continue to be underrepresented in scientific research even though the National Institutes of Health (NIH) Revitalization Act of 1993 directed the inclusion of women in human research studies unless a compelling rationale establishes that inclusion is not appropriate (Clayton and Collins, 2014; Stachenfeld, 2018). OCP users are commonly excluded in fluid balance studies due to the challenges that arise in controlling for the timing of prescription dosing, types of OCP, and the increased circulating exogenous hormone concentrations. However, with > 90% of American and European women either taking or previously taken OCPs, excluding OCP users as research participants should not be accepted as a compelling reason regardless of the justification provided. While the authors acknowledge and agree that challenges exist in including OCP users, their exclusion fails to address female specific physiology.

Hydration status may affect sex hormone impact on fluid balance. During dehydration in OCP users, Stachenfeld et al., found that combined estrogen and progestin administration increased thirst simulation and osmotically induced AVP (Stachenfeld et al., 1999). Despite these findings, there was no change in body fluid regulation during dehydration nor *ad libitum* rehydration, reaffirming that estrogen shifts body water regulation to a lower plasma osmolality operating point regardless of progestins (Stachenfeld et al., 1999). Using a previously established urine osmolality of >600 mosm/kg and the equivalent copeptin value of >6.1 pmol/L in women as a cutpoint between euhydrated and hypohydrated our mean values suggest that participants were euhydrated (Enhörning et al., 2019). While there are not established reference values, others have used a cutpoint of <13.1 pmol/L to denote non-water deprived individuals (Keller et al., 2010). Our participants were below this cutpoint and we would expect to see much greater values in a hypohydrated state. The participants in our study remained euhydrated for the duration of our trials, likely offsetting changes to thirst or copeptin response induced by a decrease in plasma osmolality. Therefore, in a euhydrated, resting state, exogenous hormones seem to have little impact on the AVP or copeptin responses to a water bolus (Stachenfeld et al., 1999; Giersch et al., 2020).

The renin-angiotensin-aldosterone system (RAAS) maintains arterial blood pressure, fluid balance and electrolyte balance. When progesterone is abundant, as in the luteal phase, Na^+^ reabsorption at the distal nephron is inhibited due to progesterone mediated inhibition of aldosterone (Myles and Funder, 1996; Stachenfeld et al., 1999). In order to compensate for natriuresis, the RAAS compensates with increased plasma renin activity and circulating plasma Na^+^ increases (Myles and Funder, 1996; Stachenfeld et al., 1999). In agreement with the findings of Stachenfeld et al. (1999), plasma Na^+^ levels did not differ between OCP phase, suggesting that exogenous forms of progestins may not act on RAAS in the same manner as endogenous progesterone (Sims et al., 2008).

Another challenge in including females in research studies is the timing of the menstrual cycle. If study methodology requires testing during a single menstrual phase, scheduling must be done a month in advance. This creates an extended timeline needed for data collection completion. Fortunately, our data suggest that different phases of OCP use do not affect the physiological responses to a fluid bolus in euhydrated, resting conditions; meaning there is no need to plan around dosing differences in OCP users if using similar study environments.

If females are more often included as research participants, there may be an increased possibility to ameliorate hydration recommendations for the female population. However, if continually excluded as research participants solely for being on OCPs, scientific literature fails to serve a large population. Previous research suggests that it is unlikely that female sex hormones and fluctuating concentrations negatively affect fluid balance during exercise (Giersch et al., 2021). Considering the ameliorated physiological stressors (euhydrated, resting conditions) in the present study it is not surprising that OCP phase does not affect physiological responses to a water bolus.

Like previously mentioned, the volume of water bolus provided in our study was based on previous data (Claybaugh et al., 2000). The amount we chose was a reasonable amount that a person would consume in the time allotted and would not maximally dilute urine. Claybaugh et al. (2000) suggested that free water losses are greater in eumenorrheic females in both the luteal and follicular phase compared to males. Although we do not have a male comparison group, both our data and data published by Claybaugh et al. (2000) show that water turnover is not different between hormone phases in both normal menstruating females and OCP users. Sollanek et al. (2018), have previously published a similar protocol using 1L of beverage to assess the hydration capabilities of other beverages compared to water. It is possible a larger fluid bolus of 1L compared to our average of 471 ml would have evoked greater changes in the assessed measures, specifically regarding the percentage of bolus excreted and frequency of voids.

The lack of baseline measures for heart rate, mean arterial pressure, perceived thirst, and urgency to void presented in Table 3 is a limitation. However, fluid consumption and food consumption for 24-h leading into the trials were matched. Urinalysis for 24-h preceding trials, including 24-h volumes, USG, and urine osmolality were not different prior to the consumption of a water bolus. Spot urine samples including USG and urine osmolality were no different prior to the consumptions of a water bolus. Blood markers of hydration (plasma osmolality and copeptin) were also no different at baseline. 15-min post-fluid ingestion there were also no differences between trials in any of the assessed variables. Therefore, despite the lack of baseline values, we do not believe this would change our findings. Interpretation of the data should consider this limitation.

Limitations prevented us from including eumenorrheic females in our study. In order to make the comparison between the normal female menstrual cycle and exogenous hormones in OCP users, we would have ideally recruited a matched sample size. However, recruiting a sample of eumenorrheic females in a university setting proved difficult in the time allowed for study execution. Although this is a limitation, we believe that the current study is representative of the large number of females using OCPs. In order to truly dichotomize phases of OCP use, we would have needed a “washout” period. Understandably, this was not feasible within our sample. Therefore, the terminology used to compare OCP phases within the present study is limited and should not be interpreted as OCP use versus no OCP use. Rather, we chose the study design to be representative of a university setting and the associated recruiting pool and to demonstrate that OCP use, regardless of dosing phase, should not be used as exclusion criteria in euhydrated, fluid balance research. We recognize that 17 subjects provide a small sample size and may contribute to a lack of significance due to a lack of power. The variability (dosing) of differing brands of OCP may also contribute to the lack of significant differences throughout our measured variables. However, this is the first study, to our knowledge, that aims to identify the responses to a water bolus in differing hormone profiles of OCP users. We encourage continuing research in this area especially with larger sample sizes to be able to extrapolate our findings.

## Conclusion

While sex hormones are known to affect fluid balance, this study documents that the hormone fluctuations between the HH and LH phase of OCP do not affect the response to an acute water bolus in euhydrated resting conditions. Further, researchers should consider these findings in their methodological design. Despite drastic fluctuations in exogenous hormone concentrations, responses to an acute water bolus seem unaffected in females who have taken combined, monophasic OCPs for >6 months. This study highlights that the exclusion of females using hormonal oral contraceptives in hydration studies may not be warranted. However, further research is needed to extend these findings to varying exercise modalities, environmental conditions, and even differing hormonal contraceptives.

## Data Availability Statement

The raw data supporting the conclusions of this article will be made available by the authors, without undue reservation.

## Ethics Statement

The studies involving human participants were reviewed and approved by the University of Arkansas Institutional Review Board. The patients/participants provided their written informed consent to participate in this study.

## Author Contributions

WA and BM conceived and designed research and analyzed data. WA performed experiments and drafted manuscript. WA, BM, AC, and SK interpreted results of experiments and edited and revised manuscript. All authors approved final manuscript.

## Conflict of Interest

The authors declare that the research was conducted in the absence of any commercial or financial relationships that could be construed as a potential conflict of interest.

## Publisher's Note

All claims expressed in this article are solely those of the authors and do not necessarily represent those of their affiliated organizations, or those of the publisher, the editors and the reviewers. Any product that may be evaluated in this article, or claim that may be made by its manufacturer, is not guaranteed or endorsed by the publisher.
